# RETRACTION: “Effectiveness and Safety of the Korean Medicine Senior Health Promotion Program Using Herbal Medicine and Acupuncture for Mild Cognitive Impairment: A Retrospective Study of 500 Patients in Seoul, Korea”

**DOI:** 10.1155/ecam/9816427

**Published:** 2025-05-15

**Authors:** Evidence-Based Complementary and Alternative Medicine

RETRACTION: H.-W. Suh, J.-H. Seol, E.-J. Bae, H.-Y. Kwak, S. Hong, Y.-S. Park, J. H. Lim, and S.-Y. Chung, “Effectiveness and Safety of the Korean Medicine Senior Health Promotion Program Using Herbal Medicine and Acupuncture for Mild Cognitive Impairment: A Retrospective Study of 500 Patients in Seoul, Korea,” *Evidence-Based Complementary and Alternative Medicine*, vol. 2021 (2021). https://doi.org/10.1155/2021/8820705.

The above article, published online on 6 December 2021 in Wiley Online Library (https://wileyonlinelibrary.com), has been retracted by John Wiley & Sons Ltd.

This article has been retracted following an investigation into concerns initially raised by the authors. Errors were identified in the published article which arose from either incorrectly performed experiments or incorrect presentation of results in the published article. These errors substantially affected both the *p* values and sample sizes in the article.

The authors requested to correct these errors, but upon assessment by the Editorial Board, it was determined that the article should be retracted due to the extent of the errors.

The authors agree to the retraction.

